# The management of trauma victims with head injury: a study by the national confidential enquiry into patient outcome and death

**DOI:** 10.1308/003588413X13511609956813

**Published:** 2013-03

**Authors:** NCE Smith, GP Findlay, D Weyman, H Freeth

**Affiliations:** National Confidential Enquiry into Patient Outcome and Death, UK

**Keywords:** Head injuries, Trauma, Peer review

## Abstract

**Introduction:**

In 2006 the National Confidential Enquiry into Patient Outcome and Death undertook a large prospective study of trauma care, which revealed several findings pertaining to the management of head injuries in a sample of 493 patients.

**Methods:**

Case note data were collected for all trauma patients admitted to all hospitals accepting emergencies in England, Wales, Northern Ireland and the Channel Islands over a three-month period. Severely injured patients with an injury severity score (ISS) of ≥16 were included in the study. The case notes for these patients were peer reviewed by a multidisciplinary group of clinicians, who rated the overall level of care the patient received.

**Results:**

Of the 795 patients who met the inclusion criteria for the study, 493 were admitted with a head injury. Room for improvement in the level of care was found in a substantial number of patients (265/493). Good practice was found to be highest in high volume centres. The overall head injury management was found to be satisfactory in 84% of cases (319/381).

**Conclusions:**

This study has shown that care for trauma patients with head injury is frequently rated as less than good and suggests potential long-term remedies for the problem, including a reconfiguration of trauma services and better provision of neurocritical care facilities.

Trauma is the leading cause of death in the first four decades of life in the UK and a leading cause of ill health.^1^ At least half of trauma cases are related to head injury^2^ and these can be either in isolation or as part of polytrauma. It therefore follows that head injury as a result of trauma can also be a major cause of long-term morbidity and a large socioeconomic burden. The chance of survival and the completeness of recovery are highly dependent on the care that follows the injuries.

For trauma patients with head injuries, timely resuscitation, avoidance of secondary brain injury and definitive operative care (where necessary) are prerequisites to optimal outcomes. An average district hospital is unlikely to treat more than one severely injured patient per week and as sufficient trauma experience cannot be achieved at all hospitals, optimal outcomes may be compromised. It has been recommended that all patients with acute severe head injuries are managed in a specialist neurosurgical/ critical care centre.^3^ Published work suggests outcome is better for those patients who are managed in a specialist centre.^4^ In 2006 a large prospective study of trauma care by the National Confidential Enquiry into Patient Outcome and Death (NCEPOD) revealed findings pertaining to the management of head injuries in a defined series of severely and often multiply injured patients.^5^

## Methods

Patients were identified prospectively from 1 February to 30 April 2006 from all hospitals accepting emergencies in England, Wales, Northern Ireland and the Channel Islands. A nominated contact in the emergency department identified all patients who were judged clinically to be ‘severely injured’. This list of patients (which included a patient identifier as well as the date and time of admission) was forwarded to a designated individual in that hospital (the NCEPOD Local Reporter). The Local Reporter returned these data to NCEPOD alongside photocopies of any documentation completed by ambulance crews at the scene of injury and on the journey to hospital together with the case notes covering the first 72 hours in hospital. Based on the case notes, the staff at NCEPOD calculated an injury severity score (ISS) for each patient. Patients with an ISS of ≥16 were included in the study.

Peer review of each case was conducted by a multidisciplinary group of advisors comprising clinicians from the following specialties: emergency medicine, anaesthetics, general surgery, intensive care medicine, maxillofacial surgery, neurosurgery, nursing, paediatrics, plastics, orthopaedics and vascular surgery. For each case reviewed, the advisor completed an assessment form, highlighting any concerns, and graded the overall care. The quality of care was categorised as:

- Good practice (a standard of care you would expect from yourself, your trainees and your institution)
- Room for improvement with aspects of clinical care that could have been better
- Room for improvement with aspects of organisational care that could have been better
- Room for improvement with aspects of clinical and organisational care that could have been better
- Less than satisfactory (several aspects of clinical and/ or organisational care that were well below a standard you would expect from yourself, your trainees and your institution)
- Insufficient data

In addition, information was requested from the admitting clinician and from the clinician responsible for the initial treatment of the patient in the accident and emergency department via a questionnaire.

To reduce any possible advisor bias, prior to any cases being seen by the advisor group, these records were anonymised not only for patient details but also the identity of the hospital and the personnel. After checking the data for face validity, a series of descriptive summaries were produced using Access^®^ and Excel^®^ (Microsoft, Redmond, WA, US). No formal hypothesis tests were planned or conducted.

## Results

A total of 795 patients met the criteria for inclusion in the study. Table 1 shows the number of patients with each possible combination of the severity of their worst head injury (as defined by the abbreviated injury scale) and the severity of their worst non-head injury. In 302 patients, there was no head injury and they were excluded from the analysis that followed, leaving 493 (366 male, 74%) for more detailed consideration. The average patient age was 30 years (range: 3–95 years). Table 2 gives a detailed breakdown of age.

**Table 1:** Study population categorised by the severity of the patient’s head injury and other most severe non-head injury. Shaded cells denote a head injury of greater severity than the worst non-head injury. Empty cells denote combinations of injury severity that would not warrant inclusion in this study.

Severity of head injury	Severity of worst non-head injury					
	None	Minor to moderate	Serious	Severe	Critical	Total
Minor to moderate			25	18	8	**51**
Serious	5	24	52	22	5	**108**
Severe	81	62	45	14	4	**206**
Critical	61	31	21	12	3	**128**
**Total**	**147**	**117**	**143**	**66**	**20**	**493**

**Table 2:** Age range

Age	Patients
0–5 years	11
6–10 years	5
11–15 years	14
16–20 years	87
21–25 years	59
26–30 years	42
31–35 years	45
36–40 years	20
41–45 years	31
46–50 years	33
51–55 years	24
56–60 years	33
61–65 years	15
66–70 years	10
71–75 years	13
76–80 years	21
81–85 years	19
86–90 years	7
91–95 years	4
**Total**	**493**

Of the 493 patients, 342 (shaded boxes in Table 1) had a head injury of greater severity than the worst non-head injury. In 147 patients, there was no other injury (isolated head injury). The predominant cause of isolated head injury was a fall from height whereas the predominant cause of head injury in those with multiple trauma was a road traffic accident. Mortality rates escalated in line with the severity of the head injury, being 8%, 11%, 15% and 31% for the increasing severity head injury groups in Table 1.

The nature of the most major component of the head injury is shown in Table 3. Many of these patients will have had a combination of scalp injury, skull fracture, cerebral contusion and intracranial haemorrhage but for clarity the most severe element of the head injuries were used to present an overall picture of the nature and relative distribution of injuries in this patient group. It can be seen that the picture is dominated by intracranial haemorrhage, a majority of which were subdural, followed by a combination of cerebral contusions and diffuse axonal injury.

**Table 3:** Predominant head injury

Severity of head injury	Predominant head injury						
	Scalp injury	Skull fracture	Cerebral contusion	Intracranial haemorrhage	Diffuse axonal injury	Other	Total
Minor to moderate	29	4	1	0	0	17	**51**
Serious	1	30	27	18	0	32	**108**
Severe	0	12	4	177	0	13	**206**
Critical	0	0	13	82	18	15	**128**
**Total**	**30**	**46**	**45**	**277**	**18**	**77**	**493**

The Glasgow Coma Scale (GCS) score on admission to hospital, broken down for each severity of head injury, is shown in Table 4. Sixty-five patients with either a serious, severe or critical head injury arrived at hospital with a GCS of 15.

**Table 4:** Study population categorised by severity of patient’s head injury and glasgow coma scale (GCS) score on admission to hospital

GCS on arrival at hospital	Severity of head injury				
	Minor to moderate	Serious	Severe	Critical	Total
15	26	17	32	16	**91**
13–14	11	19	51	18	**99**
9–12	7	13	33	10	**63**
4–8	0	31	45	39	**115**
3	3	23	37	37	**100**
**Subtotal**	**47**	**103**	**198**	**120**	**468**
No GCS recorded	4	5	8	8	**25**
**Total**	**51**	**108**	**206**	**128**	**493**

The admitting specialty was also explored among patients with multiple trauma and those with an isolated head injury. Where patients experienced multiple trauma, 68/346 patients were admitted under the trauma and orthopaedics team, 63/346 under the care of a neurosurgeon and 50/346 under the care of the critical care medicine team. Where the patient had an isolated head injury, 63/147 were admitted under the care of a neurosurgeon, 19/147 under the critical care medicine team, and 15/147 under the trauma and orthopaedics team. It important to highlight that the specialty of the admitting clinician was unknown in a large proportion of patients (129/493).

Computed tomography (CT) of the head was performed in 447 patients. In the opinion of the advisors, CT was delayed in 90/334 cases (with insufficient data to assesses in 103 cases). Examining the data from the main report, the main reasons for delay in CT were organisational issues in terms of awaiting access to CT or awaiting suitable medical staff.

Neurosurgical opinion was sought in 371 cases. Of these, 185 patients had their initial consultation with an offsite neurosurgeon and, of these, 61 had critical head injuries, 101 had severe head injuries, 21 had serious head injuries and 2 had minor to moderate head injuries. In the group of patients with the most serious head injures (severe and critical) requiring neurosurgical opinion (*n*=293), 162/274 (not answered in 19 cases) had their initial consultation undertaken by an offsite neurosurgeon. In the group of patients with isolated head injuries where a neurosurgeon was consulted (*n*=133), this consultation was undertaken off site in 75/127 cases (not answered in 6 cases).

Overall, 114 patients required neurosurgical procedures as part of their care. Of these, 45 had isolated head injuries and the remainder had multiple trauma.

In total, 58 of the 114 patients required secondary transfer to a hospital with neurosurgical capabilities for operative intervention (28 isolated head injuries and 30 multiple trauma patients). The time to surgery for patients admitted to hospitals with a neurosurgery service on site was much shorter than for those requiring a secondary transfer to a neurosurgical centre. Patients with isolated head injuries were operated on in 2.7 hours versus 9.1 hours and multiple trauma patients were operated on in 4.4 hours versus 11.7 hours.

The NCEPOD advisors’ grading of the quality of care received by these patients is shown in Table 5. To create a measure of institutional volume, the admitting hospitals were grouped in terciles according to the number of all trauma patients included in the study, the bands being chosen to form three similarly sized groups. The advisors judged there to be room for improvement (either clinical, organisational or both) in a substantial proportion of cases. The proportion of patients deemed to have received care consistent with good practice was highest for patients admitted to higher volume centres.

**Table 5:** Overall assessment of care (advisors’ opinion) versus volume of hospitals over the three-month study period

Overall assessment	Trauma workload			
	1–5 cases	6–12 cases	>12 cases	Total
Good practice	53	69	83	**205**
Room for improvement (clinical)	26	27	27	**80**
Room for improvement (organisational)	29	41	44	**114**
Room for improvement (clinical and organisational)	15	20	11	**46**
Less than satisfactory	8	9	8	**25**
Insufficient data	9	9	5	**23**
**Total**	**140**	**175**	**178**	**493**

In addition, the NCEPOD advisors provided a qualitative assessment of general head injury management. There was satisfactory management in 333 cases and unsatisfactory management in 66 cases; in 34 patients, there was insufficient data to make an assessment and in 60 cases the question was unanswered. Head injury management was explored in more detail for patients who had a neurosurgical consultation (*n*=371) and it was noted whether this consultation took place on or off site. The overall management was satisfactory in 128/145 patients where the assessment was performed on site and in 119/157 patients where the assessment took place off site. (This question was not answered or there was insufficient information in 69 cases.)

The same data were explored in more detail for the severe and critical head injury group (334 patients). For these, head injury management was satisfactory in 231 patients and unsatisfactory in 48 patients; in 55 cases, there was insufficient information to assess the overall head injury management. Again, the general management of the head injury and location of the initial consultation was explored in those patients where a neurosurgeon was consulted (Table 6). The difference in assessment of care was statistically significant (two-tailed Fisher’s exact test, *p*=0.01).

**Table 6:** Overall assessment of the management of the head injury by location of neurosurgical assessment

Overall management of head injury satisfactory?	Location of neurosurgical assessment				
	On site	Off site	Subtotal	Not documented	Total
yes	93	106	199	14	213
No	10	32	42	1	43
**Subtotal**	**103**	**138**	**241**	**15**	**256**
Insufficient data	2	13	**15**	2	**17**
Not answered	7	11	**18**	2	**20**
**Total**	**112**	**162**	**274**	**19**	**293**

## Discussion

This was a prospective study and all evaluations were made on a full exploration of the case notes as well as of the reports of the clinicians involved in care. The anonymity of the cases ensured that assessor bias was excluded as far as possible. We believe that the data and assessments are objective.

In this population of severely injured patients, head injury was very common (493/795 patients, 62%). Of these patients, 147 (30%) had isolated head injuries with the remainder suffering multiple trauma.

Current UK practice does not allow the prehospital staff to greatly influence where injured patients are taken, with the result that patients may arrive at hospitals without the necessary infrastructure to provide their definitive care. This appears to be supported by our data. Of the 371 cases where neurosurgical opinion was sought, this was undertaken off site in 185 cases. For the group with the most serious head injures (severe and critical) this figure was 162/274 (59%) and in the group of isolated head injuries (where a neurosurgeon was consulted, *n*=133) this figure was 75/127 (59%) (not answered in 6 cases). These data suggest that the current system of transfer from the scene of injury to secondary care is not delivering patients to the most appropriate hospital.

This is a major failing as outcome for patients with acute severe head injuries is better when treated in a neurosurgical centre, irrespective of the need for surgical intervention^4^ and direct admission to a centre with neurosurgical services on site (rather than admission to a local hospital followed by secondary transfer) is associated with improved survival.^6^ Trauma networks are now being established in the UK,^7^ raising the possibility of more prehospital triage and centre bypass for selected patients. However, even with such a system, triage is difficult, and both undertriage and overtriage do occur.^8^ Combined with the lack of immediate change in trauma systems across the UK, this emphasises the need for all hospitals with emergency departments to be skilled in the initial management, resuscitation and investigation of head injured patients and to have robust protocols for communication with their local specialist centre.

### Investigation

Investigation by CT allows the identification of patients who require intervention to prevent secondary brain injury. A quarter of all patients were judged not to have had timely CT (90/344, 26%) and delay was present across all head injury severity groups. Delays of this magnitude may affect outcome adversely. The National Institute for Health and Clinical Excellence has produced guidelines in this area^3^ and our data show that current practice falls far short of these guidelines. Most delays were due to organisational factors (primarily waiting for radiology staff who were non-resident to arrive at hospital to perform CT), again highlighting the problem of responding in a timely fashion to a clinical scenario that does not happen frequently.

### Admitting specialty

While neurosurgery was the most common admitting specialty for head injured patients in this study, it is clear that patients also continue to be admitted under the care of many other specialties (notably, trauma and orthopaedics, critical care and general surgery). In circumstances where a patient with a head injury requires hospital admission, it is recommended that the patient be admitted only under the care of a team led by a consultant who has been trained in the management of this condition during his or her higher specialist training.

The consultant and his or her team should have competence (defined by local agreement with the neuroscience unit) in assessment, observation and indications for imaging; inpatient management; and indications for transfer to a neuroscience unit.^3^ Given the wide variety of specialties involved and the continued management of patients with severe acute head injury in non-specialist centres, this necessitates continued education and training for a wide cohort of staff in the management of these patients.

### Head injured patients taken to non-neurosurgical centres

Despite the knowledge that outcome may be improved by treatment in a specialist centre,^4^ this frequently does not happen, as shown in our data. This NCEPOD study adds a qualitative assessment to the body of quantitative data linking poor outcome with non-specialist centre placement. Peer review of the cases, using the NCEPOD methodology, showed that all patients with head injury, either isolated or as part of multiple trauma, were more likely to receive less good care if they were managed in a non-specialist centre. For the most severely head injured patients this achieved statistical significance. Although the overall assessment of head injury management was satisfactory in a majority of cases in this group, patients admitted to a non-neurosurgical centre requiring neurosurgeon consultant review were significantly more likely not to have satisfactory management of their head injury than those admitted directly to a centre with neurosurgeons on site.

Capacity in dedicated neurosurgical units appears to be the major constraint. The Neurocritical Care Stakeholder Group estimated that in England and Wales around 4,000 patients per year were being managed in a district general intensive care unit rather than a specialist unit.^9^ It appears that a large number of head injured patients are being failed by the current system of care in the UK, and that quality of care is compromised and outcomes are adversely affected.

### Delays to investigation and definitive management

Timely surgical intervention is required for optimal patient outcomes, particularly in the group of patients with intracranial haematomas. This is clearly affected by whether the patient arrives at a specialist hospital initially or requires a secondary transfer. The average time to surgical intervention was much longer for those patients requiring secondary transfer. In addition, the proportion of patients receiving surgery within four hours of injury was substantially lower in the transferred group compared with the group with direct admission to a specialist centre (14% vs 67%).

The Brain Trauma Foundation recommends that surgery for extra and subdural haematomas should be performed as soon as possible^10^ and UK guidance advises that such procedures should be undertaken in less than four hours.^11^ Our data show that current UK practice is falling well short of these recommendations. This has been shown in another study where mean times to transfer for surgery in patients with extradural and subdural haematomas was 5.25 and 6 hours respectively.^12^ There needs to be urgent attention to strategies to reduce the time taken to reach definitive care, ensuring that avoidable delays are minimised.

## Quality of care and overall volume

In the full report, *Trauma: Who Cares?*, we point to the lack of appreciation of the severity of illness, of the urgency of the clinical scenario and incorrect clinical decision making that were apparent.^5^ Many of these clinical issues were related to the lack of seniority and experience of the staff involved in the immediate management of these patients. It was clear that the provision of suitably experienced staff during evenings and nights was much lower than at other times. In the management of trauma, which very often presents at night, this is a major concern.

Severe trauma is not common in Britain and many hospitals see less than one severely injured patient per week. This has a direct bearing on experience and ability to manage these challenging patients. Not only does this relate to clinical skills but also to the feasibility of providing the entire infrastructure required to manage trauma patients definitively in all centres. In this head injured group of patients, we have shown that higher volume hospitals have a trend towards better care.

### Conclusions

This study has shown that the care for trauma patients with a head injury is frequently less than good. Simple remediable steps in the initial care of these patients could be implemented by individual hospitals and trusts using published guidelines,^3,10,11^ and their use could be self-audited. The National Center for Injury Prevention and Control in the US suggests that 80% compliance with the Brain Trauma Foundation guidelines would not only save 3,000 lives per year but also $250 million per year in reduced medical and rehabilitation costs.^13^ Longer term actions that will improve the care (and outcome) of these patients include a robust reconfiguration of trauma services and better provision of neurocritical care facilities.
